# Notes and News

**Published:** 1905-11-11

**Authors:** 


### Notes and News.

The next French Congress of Medicine will meet in Paris under the Presidency of Professor Debove.

The Queen has subscribed £100 towards the memorial to be erected to Finsen, in Denmark. The King has also subscribed, and the collection now amounts to 100,000 crowns.

A lady (Miss Jane Woollen) has been appointed as house-steward and assistant dispenser at the General Hospital, Tasmania. She was for five years head dispenser at the Queen's Hospital, Melbourne.

It will be interesting to know whether the self-levelling bunks which are to be adopted on the cross-Channel boats will really alleviate some of the misery which those prone to sea-sickness now suffer. Those bunks which have been used experimentally are said to have been successful.

By the courtesy of the House Committee of the Westminster Hospital, the annual meeting of Presidents of the League of Mercy will be held in the board-room of that hospital on Friday, December 15, at four o'clock, when the chair will be taken by the Right Hon. the Earl of Mansfield, President of the North St. Pancras district.

Dover has lately become one of the first favourites as a seaside health resort. Medical men are now recommending it during the summer and early autumn as being more bracing than many places on the South Coast and less so than some on the East Coast. When the harbour is completed and it becomes more naval in character, it will certainly become more popular. At present there is a scare amongst householders on the question of rates, and it is said that there are eight hundred empty houses.

We regret to learn that on the 4th inst. the premises of Messrs. John Wright and Co., of Bristol, the well-known firm of publishers, were completely destroyed by fire.

The war against quackery in medicine is becoming very pronounced in Europe. In Austria a society has been formed for the suppression of quackery, and a French congress for the same purpose will be held in Paris next year.

Mr. John Henry Johnson has been appointed Secretary to the Royal Westminster Ophthalmic Hospital, in succession to Mr. T. Beattie Campbell, who has retired on a pension after twenty-one years' service.

The medical profession in Austria has suffered so much through the sickness and insurance clubs that the Union of the Medical Chambers has taken up the matter with a view to limiting the formation of these societies, and creating a scale of payment according to means.

The Parliamentary Sub-Committee of Manchester recommend the City Council to seek further powers for sanitary purposes. The suggestions they have made are excellent, and include a recommendation that the vendors of ice-creams and shellfish should have their names and addresses written conspicuously on their barrows.

The Chelsea Hospital for Women has received from the Merchant Taylors' Company a donation of £31 10s. towards the expenditure recently incurred to give the patients the benefit of recent medical science. The Hon. Mrs. Lionel Tollemache, to supplement this work, has given £50 to the Convalescent Home.

On Friday, November 3, Sir Frederick Treves paid a visit to the General Hospital, Tunbridge Wells. He was met by the President (Marquis Comden) and the Treasurer (F. W. Elers, Esq.), and was escorted round the hospital accompanied also by Mr. Lammiman (one of the consulting surgeons), the house surgeon, and the matron. A visit was first made to the Out-Patient Department and Dispensary, and from thence to the wards, each of which came in for its share of praise. But, as was only natural, the operating theatre attracted Sir Frederick most of all, and he expressed the opinion that, "It could not be better." At the meeting of the Surgical Aid Society afterwards, Sir Frederick said that the Tunbridge Wells Hospital was perfect in all its appointments.

We note that the Committee of the hospital have just received munificent offers from two donors of £1,000 each, provided another £2,000 is raised by January 31, 1906, to help pay off the deficit on the Building Fund. A great effort is being made to fulfil the necessary condition so that the offers may be accepted.

